# Thermal performance of fish is explained by an interplay between physiology, behaviour and ecology

**DOI:** 10.1093/conphys/coz025

**Published:** 2019-06-10

**Authors:** Philipp Neubauer, Ken H Andersen

**Affiliations:** 1 Dragonfly Data Science, Level 4, 158 Victoria St., Stephenson & Turner House Te Aro, Wellington New Zealand; 2 Centre for Ocean Life, National Institute of Aquatic Resources, Technical University of Denmark, 7 Kemitorvet B 202, Kongens Lyngby, Denmark

**Keywords:** Climate change, metabolic rate, OCLTT, optimal foraging, thermal performance

## Abstract

Increasing temperatures under climate change are thought to affect individual physiology of fish and other ectotherms through increases in metabolic demands, leading to changes in species performance with concomitant effects on species ecology. Although intuitively appealing, the driving mechanism behind thermal performance is contested; thermal performance (e.g. growth) appears correlated with metabolic scope (i.e. oxygen availability for activity) for a number of species, but a substantial number of datasets do not support oxygen limitation of long-term performance. Whether or not oxygen limitations via the metabolic scope, or a lack thereof, have major ecological consequences remains a highly contested question. size and trait-based model of energy and oxygen budgets to determine the relative influence of metabolic rates, oxygen limitation and environmental conditions on ectotherm performance. We show that oxygen limitation is not necessary to explain performance variation with temperature. Oxygen can drastically limit performance and fitness, especially at temperature extremes, but changes in thermal performance are primarily driven by the interplay between changing metabolic rates and species ecology. Furthermore, our model reveals that fitness trends with temperature can oppose trends in growth, suggesting a potential explanation for the paradox that species often occur at lower temperatures than their growth optimum. Our model provides a mechanistic underpinning that can provide general and realistic predictions about temperature impacts on the performance of fish and other ectotherms and function as a null model for contrasting temperature impacts on species with different metabolic and ecological traits.

## Teaser

Although a number of theories exist, there is currently little consensus on drivers of thermal performance in ectotherms, and fish in particular. We present a general size and trait based model that shows that thermal performance can be explained by an interplay of physiological, ecological, and behavioural constraints.

## Introduction

Temperature, through its effects on individual physiology, is a dominant driver of species ecology and biogeography (Pinsky *et al*., 2013; Deutsch *et al*., 2015). As a consequence, current and predicted temperature increases under climate change will act as a strong agent of change in many ecosystems (Walther *et al*., 2002; Parmesan and Yohe, 2003; Deutsch *et al*., 2015; Stuart-Smith *et al*., 2015). Such predictions of changes in species ecology based on physiological function are important to ensure appropriate policy and management response to changing environments and expected effects on organisms (McKenzie *et al*., 2016; Patterson *et al*., 2016). However, the nature of these changes can be difficult to predict as temperature effects scale from individuals to species and ecosystems. Through this cascade of scales, incorrect or approximate model assumptions at the individual scale can have disproportionate effects on ecosystem-level outcomes (Brander *et al*., 2013; Lefevre *et al*., 2017). In marine fish, for example, recent models suggest decreasing organism size with warming temperatures under climate change, and resulting decreases in the size of fish that make up fisheries catches (Cheung *et al*., 2013). Such changes in fish growth and size would have downstream implications for fisheries stock assessment (e.g. by changing population productivity) and management tools such as size limits. However, these predictions have been criticized as overly simplistic and not in line with physiological constraints (Brander *et al*., 2013; Lefevre *et al*., 2017).

Although there are conceptual and model frameworks to explain aspects of thermal performance and ecological responses to temperature (Fry, 1947; Brown *et al*., 2004; Pörtner, 2010; Pauly and Cheung, 2017), many of these remain controversial as they appear limited in their generality or predictive capacity (Lefevre *et al*., 2017; Jutfelt *et al*., 2018). To our knowledge, no general theoretical framework exists to quantitatively explain and predict changes in ecological rates, such as observed change in growth and asymptotic size (e.g. the temperature-size rule in ectotherms; Atkinson, 1994; Angilletta *et al*., 2004), or attack rates (Englund *et al*., 2011; Rall *et al*., 2012) with temperature, from fundamental physiological processes. Instead, and as advocated by the metabolic theory of ecology (Brown *et al*., 2004), ecological theory often treats ecological rates as being directly temperature dependent, without a direct link to the underlying physiological drivers (Angilletta *et al*., 2004; Vucic-Pestic *et al*., 2011; Guiet *et al*., 2016).

A phenomenological description that assumes a general ecological temperature response often fails to explain heterogeneity in ecological responses (Angilletta *et al*., 2004; Rall *et al*., 2012). Although ecological rates seem to follow some general patterns in the response to temperature, there is also significant heterogeneity between species and trait groups (Angilletta *et al*., 2004; Englund *et al*., 2011; Rall *et al*., 2012). This leads to difficulties with extrapolation across species or other model components (Guiet *et al*., 2016). In this case, a deeper understanding of the underlying drivers of thermal responses may be necessary in order to derive general predictions about ecological responses to changing temperatures (Vucic-Pestic *et al*., 2011; Lefevre *et al*., 2017). Since the primary effect of temperature on organisms is on individual physiology, a general model to explain ecological response should be grounded in physiology.

Physiologically, a long-held view has been that temperature is a controlling factor while oxygen supply sets the physiological limits (Fry, 1947; Claireaux and Lefrançois, 2007; Lefevre, 2016). How exactly temperature influences ectotherm physiological rates and limits, however, has been a matter of debate, not least because of the variable responses observed among different species. In most species, the standard metabolic rate (SMR; the metabolic cost of maintenance and routine activity such as ventilation) increases near exponentially with temperature. A prevalent view is that the maximum metabolic rate (MMR; the metabolic rate at maximum sustained exercise) has a dome-shaped response to temperature, whereby it can be increased (passively and actively) up to a point, but plateaus or decreases thereafter (Fry, 1947; Claireaux and Lefrançois 2007; Pörtner and Farrell, 2008; Lefevre, 2016). This leads to the view of a unimodal curve for metabolic scope (MMR minus SMR; the available oxygen/energy for additional activity) and suggests that towards the upper end of this curve, organisms will, simply put, run out of oxygen.

This view was encapsulated in the theory of oxygen and capacity limitation of thermal tolerance (OCLTT; Pörtner, 2010), which suggests that the decrease in metabolic scope towards extreme temperatures limits species’ ability to sustain core functions such as foraging and growth (i.e. functions beyond SMR). In some species, however, MMR increases steadily (Lefevre, 2016; Verberk *et al*., 2016), suggesting that oxygen may not be the limiting factor at high temperatures. Indeed, it has been argued that oxygen is unlikely to determine performance for most species over most of their temperature range as oxygen limits are rarely reached during normal activity (Holt and Jorgensen, 2015; Jutfelt *et al*., 2018).

Here, we propose a quantitative size- and trait-based ecophysiological model to derive general predictions about temperature impacts on fish physiology, performance and ecology. We describe simple size-dependent physiological processes within an ecological context, and, using a simple optimization argument, show that observed ecological responses of different life-history strategies can be predicted on the basis of optimized bioenergetics under different temperatures.

## Methods

### Key assumptions

Our model assumes that physiology is described by two key budgets: the energy and oxygen budgets (Holt and Jorgensen, 2014, 2015). We assume that animals will adapt activity levels to optimize available energy for growth and reproduction relative to mortality risk. Available energy is determined either by food capture, by food processing capacity or by available oxygen. We further assume that temperature acts directly on rates that are determined by enzymatic activity: digestive activity (via maximum consumption) and metabolic costs. Consequently, temperature only acts on ecological rates (e.g. actual feeding rates) via optimization of activity levels.

### Model description

Ectotherms adjust the relative amounts of time (τ) spent on metabolically costly activity and resting/hiding to optimize the net energy gain relative to mortality (Gilliam and Fraser, 1987). In the following, we refer to τ as the activity fraction for sake of generality. Since both energy gain and metabolic losses are sensitive to temperature and oxygen limitations, both the activity level and the net energy gain will be subject to these environmental constraints. Their interplay thus determines available energy for growth and reproduction.

Net energy gain *P* (mass per time) is the difference between supply *S* and metabolic demands *D*, each being functions of body weight *w* and temperature *T*:

(1) \begin{equation*} P\left(w,T,\tau\right)=S\left(w,T,\tau\right))-D\left(w,T,\tau\right)) \end{equation*}

(2) \begin{align*} =\left(1-\beta -\phi \right)f\left(w,T,\tau \right) hc(T){w}^q\nonumber\\-c(T){kw}^n-\tau c(T){k}_aw .\end{align*}

For supply, $hc(T){w}^q$ is the maximum consumption rate, and *f*(*w*,*T*,τ) is the activity-dependent (i.e. a function of τ) feeding level as a fraction between 0 and 1. The supply is discounted by the loss due to specific dynamic action β (SDA, or heat increment; the energy spent absorbing food), and φ is the fraction of food excreted and egested.

The feeding level is given by a Holling type II functional response:

(3) \begin{equation*} f\left(w,T,\tau \right)=\frac{{\tau \gamma \varTheta w}^p}{{\tau \gamma \varTheta w}^p+ hc(T){w}^q}. \end{equation*}

The feeding level is therefore determined by the fraction of time spent foraging τ (henceforth the activity fraction), foraging rate ${\gamma w}^p\varTheta$ (search rate ${\gamma w}^p$ times food resource availability $\varTheta$) and maximum consumption $hc(T){w}^q$.

Metabolic demands (*D*(*w*,*T,τ*)) are standard metabolism (SMR; $ {kw}^n$), which scales with exponent *n* < 1, and active metabolism $ {\tau k}_aw$, which scales proportional to mass owing to muscular demands scaling approximately isometrically with weight (Brett, 1965; Glazier, 2009) and the activity fraction. Temperature scaling of metabolic rates (standard and active metabolism and maximum consumption rate) is determined by enzymatic processes (e.g. digestion, glycolysis; Jeschke *et al*., 2002; Sentis *et al*., 2013) and approximated by an Arrhenius scaling $c(T)={e}^{E_a(T-{T}_0)/(b T {T}_0)}$ (Gillooly *et al*., 2001), where ${E}_a$ is the activation energy, assumed constant, ${T}_0$ is the reference temperature (such that c(T) = 1 at 15°C) and *b* is the Boltzmann constant. Note that we only scale rates related to enzymatic activity with temperature, we do not assume that ecological rates such as foraging rates or activity are a direct function of temperature. Rather, they are modulated by an individual’s behavioural response to temperature-driven physiological changes.

The oxygen budget ${P}_{O_2}(w,T,\tau)$ (or aerobic scope) follows a similar form to the mass budget

(4) \begin{equation*} {P}_{O_2}\left(w,T,\tau\right))={S}_{O_2}\left(w,T, \tau\right))-{D}_{O_2}\left(w,T,\tau\right)) \end{equation*}

(5) \begin{align*} &\;={S}_{O_2}(T){w}^n-\omega c(T)\nonumber\\ &\qquad\left(\beta f\left(w,T,\tau \right){hw}^q+{kw}^n+{k}_aw\right) .\end{align*}

**Figure 1:**
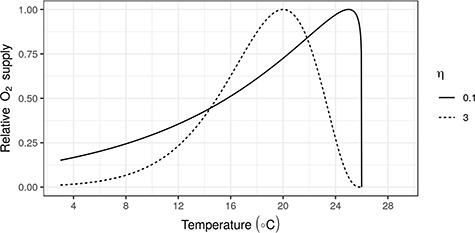
MOS relative to the maximum supply for species with a dome-shaped MOS (here η = 3) and a continually increasing MOS (η = 0.1) used in model scenarios discussed below.

Demand (${D}_{O_2}(w,T,\tau)$) is the sum of oxygen used for all metabolic processes (except assimilation losses), with ω being amount of oxygen required per mass. The oxygen supply (${S}_{O_2}(w,T,\tau)$) scales with body weight as ${w}^n$ multiplied by a flexible dome-shaped function that can emulate both a dome-shaped maximum oxygen supply (MOS) as well as a MOS that increase continuously up to a lethal temperature (Fig. 1). The maximum oxygen consumption is the oxygen consumption during maximal activity level that can be sustained over some time and corresponds to the MMR in our model. Although the MMR is often used synonymously with both MOS and demand, in some species the maximal oxygen consumption (${D}_{O_2}^{max}$) is not reached at maximum activity levels, but rather during digestion (Priede, 1985). In our model, MMR and MOS are equivalent as we do not explicitly model contributions from anaerobic metabolism, such as during burst swimming or hypoxia. During such events, the actual metabolic rate may be higher than oxygen consumption alone would suggest; however, such states cannot usually be sustained (and therefore fall outside of the sustained MMR defined here). We assume that oxygen supply, taken as the aggregated process of oxygen delivery from diffusion across respiratory organ membranes (e.g. gills) to delivery for cellular metabolism, is temperature dependent and follows a flexible dome-shaped function (Lefrancois and Claireaux, 2003; Gnauck and Straškraba, 2013):

(6) \begin{equation*} {S}_{O_2}(T)=\lambda (T)\left(1-{e}^{\left({C}_{O_2}(T)-{C}_{O_2}^{crit}\right)\log (0.5)/\left({C}_{O_2}^{50}-{C}_{O_2}^{crit}\right)}\right) \end{equation*}

(7) \begin{equation*} \lambda (T)=\zeta {\left(\frac{T_{\mathrm{max}}-T}{T_{\mathrm{max}}-{T}_{opt}}\right)}^\eta \exp \left(- n\frac{T_{\mathrm{max}}-T}{T_{\mathrm{max}}-{T}_{opt}}\right) .\end{equation*}

Here λ(*T*) specifies the temperature dependency of ${O}_2$ supply, whereas the second term in ${S}_{O_2}(T)$ term describes the dependence on ambient ${O}_2$ concentrations at temperature $T$ (${O}_2(T)$). At constant temperature $T$, oxygen supply is a function of ambient oxygen and is assumed to follow a saturating function (Lefrancois and Claireaux, 2003). We specify ${C}_{O_2}^{50}$ as the point where oxygen supply has dropped by 50% relative to the saturation level λ(*T*), and ${C}_{O_2}^{crit}$ is the ambient concentration at which oxygen supply ceases. Ambient oxygen concentration levels are assumed to decline with temperature according to a curve that approximates declines of dissolved oxygen in saltwater at 35 PSU as $l\cdot {e}^{-0.01851 (T-5)}$, with *l* the oxygen concentration at 5°C. To specify λ(*T*), we define ${T}_{max}$ as the lethal temperature for the species, and ${T}_{opt}$ as the temperature at which oxygen supply is maximized; η determines the width of the dome shape and ζ its height. Note that the simulated increase in the aggregated oxygen supply includes potential increases in oxygen delivery via increased diffusive (passive) supply at higher temperatures (Verberk *et al*., 2011) as well as increased active delivery of oxygen made possible by increased heart rates at higher temperatures (Lefrancois and Claireaux, 2003). With the above formulation, we can emulate an oxygen supply (and hence MMR) that increases up to the lethal temperature by setting the temperature for maximum oxygen delivery close to the lethal temperature (Fig. 1).

In our model, fish will adjust their activity fraction τ to maximize fitness. We use Gilliam’s rule as a fitness proxy Gilliam and Fraser, 1987:

(8) \begin{equation*} \tau ^\ast =\textrm{argmax}_{\tau}\left\{\frac{P\left(w,T,\tau\right))}{M(w,\tau)}\right\}. \end{equation*}

This optimization represents a ‘short-sighted’ fitness optimization that does not account for future changes in conditions and is appropriate for optimization in stable environments (Sainmont *et al*., 2015–09). Mortality scales with activity fraction and weight as ${w}^{q-1}$ (Andersen *et al*., 2009; Hartvig *et al*., 2011):

(9) \begin{equation*} M(w,\tau)=\left(\rho +\mu \tau \right){w}^{q-1}. \end{equation*}

In this equation, ρ is the base mortality at mass *w* = 1 and τ = 0, that is, with no activity beyond that covered by standard metabolism, and μ is the coefficient for activity-related mortality. By adjusting feeding activity τ, fish therefore simultaneously modulate their potential food intake, mortality risk and metabolic costs of activity. Activity is limited by available oxygen, such that the aerobic scope ${P}_{O_2}$ is not allowed to be negative over the timescales considered. In other words, we consider timescales that are long enough to ignore the ability of many ectotherms to go into oxygen debt or to switch to anaerobic metabolism for limited periods of time. This means we assume that animals will adjust their foraging effort to optimize fitness given temperature and oxygen constraints. Note that this optimal foraging assumption drives ecological responses as a consequence of physiological constraints, rather than as a direct response to temperature itself.

That fish behave optimally to maximize food acquisition relative to mortality risk and energetic requirement is a standard hypothesis and assumption in ecological models (Priede, 1977; Gilliam and Fraser, 1987; Claireaux *et al*., 2000; Hufnagl and Peck, 2011; Sainmont *et al*., 2015–09). Indeed, heightened mortality risk may be a key driver of fish spending very little time *in vivo* at metabolic regimes that approach the MOS (Priede, 1977).

### Defining performance metrics

Performance itself is a vague concept that is often used without definition in the relevant literature, but it is only defined in the context of a particular physiological or demographic parameter, with potentially different thermal response curves (Jutfelt *et al*., 2018). For instance, even though growth in ectotherms is often impacted by temperature (Angilletta *et al*., 2004), measured performance may depend strongly on what aspect of growth is measured—whether it is the growth increment at a particular size, growth efficiency per unit intake, the attained asymptotic size or parameters of a fitted growth curve. We use and compare four performance measures with broad ecological and practical implications:

• Growth curves predicted through ontogeny, which allow us to compare growth performance early and late in life (i.e. juvenile growth versus asymptotic size).

• Production efficiency, here defined as available energy for both growth and reproduction, relative to the food consumption (i.e.$\frac{P(w,T,\tau)}{f(w,T,\tau ) hc(T){w}^q}$).

• The dimensionless ratio of $\frac{P}{Mw}$, which is analogous to the short-sighted fitness approximation (Gilliam’s rule) used above.

• Fitness integrated over an individual’s lifetime, defined as ${R}_0$ (see below).

### Growth response

Temperature affects growth via its effects on the energy budget and the investment of available (surplus) energy into reproduction and growth. The change in allocation to reproduction with size and age in variable environmental conditions is described by the maturation reaction norm (MRN), which is generally defined as the probability of maturing at a certain age under different growth conditions (Dieckmann and Heino, 2007). We defined the reaction norm as the mid-point of a logistic allocation function that determines investment in reproduction as a function of age and size.

The slope of MRNs is evolutionarily determined by the strength of the covariation between growth and mortality for a given population in a given environment. Strongly positive covariation leads to the relatively flat reaction norms observed for most fish populations (Marty *et al*., 2011). This covariation is probably the consequence of good growth conditions (e.g. from increased food resources) altering the baseline mortality ρ and the risk of foraging μ (e.g. by attracting predators). We did not explicitly model these interactions here (aside from the dependence of mortality on τ), but rather assumed that reaction norms evolved over regimes of relatively stable temperature and growth variation in the past. Consequently, we assumed that the evolved slope of the reaction norm is a fixed trait over the timescales considered here for a particular species or population, and for simplicity and generality we assume a flat reaction norm (i.e. allocation to reproduction is a function of size only; Fig. S1), although sloping reaction norms can be formulated and used in our framework (see Appendix 1). The intercept of this reaction norm was found numerically by maximizing fitness (${R}_0$, see below) at the reference temperature for our simulations (15°C), and this intercept was assumed fixed as temperatures change.

The allocation was parametrized as

$$ \phi \left({w,w}^{\ast}\right)=1/\left(1+\exp \left(-c(w-w^{\ast})\right)\right), $$

where ${w}^*$ is the intercept of the reaction norm, and *c* determines how rapidly energy allocation shifts from somatic growth to reproduction.

As growth is also fundamentally driven by resource availability, we contrast the growth response to temperature at the baseline level (Table 1), with a 1/3 reduction and increase in available resources.

**Table 1:** Parameters of the constrained activity model for two scenarios: slow strategy and fast strategy species.

Description	Symbol (unit)	Value	
		Slow strategy	Fast strategy
**Biomass metabolism**			
SDA	β	0.15	
Egestion and excretion	φ	0.25	
Coeff. for std. metabolism	*k* (g$^{1-n}$·y $^{-1}$)	1	1.5
Coeff. for act. metabolism	*k* $_{a}$(g·y $^{-1}$)	4	2
Exponent for std. metabolism	*n*	0.88	0.75
Feeding ecology			
Coeff. for encountered food	γΘ (g$^{1-p}$·y $^{-1}$)	60 (40/80)	
Exponent for clearance rate γ	*p*	0.8	
Coeff. for maximum consumption rate	*h* (g$^{1-q}$·y $^{-1}$)	30	60
Exponent for max. consumption *h*	*q*	0.8	
Coeff. for constant mortality	ρ (g·y$^{-1} $)	0.1	1
Coeff. for activity-related mortality	µ(y$^{-1} $)	6	1
**Temperature**			
Reference temperature	(°C)	15	
Activation energy		0.52	
Temperature at maximum MOS		20/25	
Temperature range	–	5–26	
**Reaction norm**			
Slope		0	
Reaction	*c*	0.5	
**Oxygen budget**			
Critical	(mg·L$^{-1} $)	2	
Dissolved at	(mg·L$^{-1} $)	4	
Doming for supply	η	3/0.1	
Level of supply	ζ (g·y$^{-1} $)	0.5	1

For parameters with dual values (i.e. x/y), the former reflects species with a domed MOS with respect to temperature, whereas the latter corresponds to species with a continuously increasing MOS.

Values in brackets for γ are alternative resource availability scenarios.

### Fitness consequences

Fitness consequences for particular life-history strategies (trait combinations, see below) at different temperatures can be investigated if one considers the timescales in the model to be short relative to evolutionary timescales (i.e. if the model represents ecological timescales). On these timescales, we assume that adaptive responses are negligible (but see Sandblom *et al*., 2016; Moffett *et al*., 2018). We investigate overall fitness with respect to temperature by calculating, the lifetime reproductive output, for a given MRN and trait parameters—we thus do not consider evolutionary consequences of changes in fitness here. ${R}_0$ is the appropriate measure of fitness when density dependence mainly operates early in life (Kozlowski *et al*., 2004), as is often assumed for fish (Andersen *et al*., 2017; Lorenzen and Camp, 2018) and was calculated as

(10) \begin{equation*} {R}_0(T)={\int}_0^{\infty}\phi \left(w(t),{w}^{\ast}\right)P\left(w(t),T,\tau\right){S}_{0\to t}(T) dt, \textrm{where} \end{equation*}

(11) \begin{equation*} {S}_{0\to t}(T)=\underset{0}{\overset{t}{\int }}\exp \left(-M\left(w(t),\tau\right)\right) dt \end{equation*}

${S}_{0\to t}(T)$ is survival to age *t*, which is found by integrating over instantaneous survival ($\mathit{\exp}(-M(w(t),\tau))$) from age zero to *t*, where *M* depends on weight-at-age (*w*(*t*)) and temperature *T* via temperature-driven activity. Fitness is the integral over energy allocated to reproduction at age *t* and corresponding weight *w*(*t*), $\boldsymbol{\phi} (w(t),{w}^*)P(w(t),T,\tau)$ (the reproductive output) and the probability of surviving to age *t*.

### Trait-based scenarios

To ensure a level of generality beyond existing, species-specific ecophysiological models, we explored ecological impacts of optimized behaviour at different temperatures in a trait-based context. In doing so, we hoped to bridge the existing gap between detailed species-specific models, and general, largely conceptual theory describing temperature impacts. Specifically, we contrast species along a gradient of life history that, at the one end, maximizes production (energy acquisition; henceforth called the fast strategy, indicated by a subscript *f*) at the cost of increased metabolism and mortality, and at the opposing end minimizes mortality and metabolic costs at the expense of production (henceforth slow strategy, indicated by a subscript *s*). This axis leads to an approximately constant ratio of production to mortality and corresponds to a line of equal size in the life-history space proposed by Charnov *et al*. (2013). In other words, this axis contrasts species of similar size (here ${L}_{\infty}\sim 30$cm or ${w}_{\infty}\sim 270$g) with defensive/sluggish versus active life histories.

**Figure 2:**
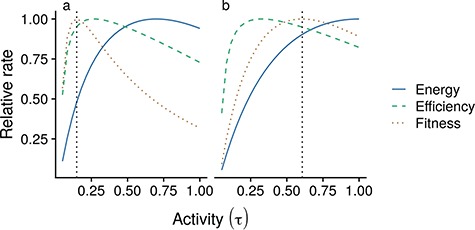
Available energy *P* (Blue solid line), efficiency (green dashed line) and the ratio of *P* to *M* (orange dotted line) are impacted as a function of changing activity at constant temperature (*T* = 15°C) for a growing fish (cm) at 10 cm length (10 g). Responses are shown for slow (a) and fast strategy (b). All rates are plotted relative to their maximum for each trait scenario; the optimal activity level is indicated by the dotted vertical line.

To implement this axis, we used the result that species with a more active, production-oriented life history (e.g. predatory pelagic fish) have a higher standard metabolism and lower weight scaling of metabolic costs (Priede, 1985; Killen *et al*., 2010). We assumed that higher standard metabolism is due to increased digestive capacity (i.e. is used for gut maintenance), though high muscle mass and a larger heart will also contribute to higher standard metabolism in active species (Priede, 1985). In practice, we assumed that ~50% of the standard metabolic cost is due to supporting organs associated with feeding activity alone, such that a doubling of the maximum ingestion leads to a 50% increase in standard metabolic cost. We further assumed that such active species have a less effective refuge from predators and therefore have a higher constant mortality, but lower mortality related to activity (i.e. ${M}_s(w)=(0.1+6\tau ){w}^{q-1}$ and ${M}_f(w)=(1+\tau ){w}^{q-1}$). Exact parameter values for these trait scenarios are given in Table 1. Together, these assumed trait differences lead to very different ecological and bioenergetic responses of slow and fast strategists (Fig. 2), with ${\tau}^*$ found at lower activity levels for slow strategists, as high activity induces exceedingly high mortality and decreasing energy efficiency (i.e. available energy relative to food intake) at high activity levels. A slower increase in available energy and M with τ for fast strategists leads to a higher ${\tau}^*$.

We further contrasted species with oxygen limitation at high temperatures (i.e. species with a unimodal metabolic scope) with species that do not experience oxygen limitation at high temperatures (at least not up to a lethal temperature, where death may be induced by sudden failure to deliver oxygen to vital organs, or failure of biochemical pathways at high temperature (Iftikar and Hickey, 2013; Salin *et al*., 2016)). In practice, this was achieved as described above by setting the maximum oxygen delivery close to the lethal temperature (Fig. 1). Note that, although we assume here that limitations over the temperature range are due to oxygen availability, other limiting mechanisms, such as the respiratory control ratio (Iftikar and Hickey, 2013; Salin *et al*., 2016), may determine upper limits to activity over some or all of a species temperature range. However, the overall mechanism would be the same to the one assumed here, with different units (e.g. ATP instead of ${O}_2$).

Our scenarios were parametrized to allow for excess metabolic scope beyond maximum foraging activity (i.e. τ = 1). This assumption is in line with observations that the aerobic scope often exceeds energetic requirements from swimming alone and is adapted to provide oxygen for digestion (SDA), the oxygen demand of which can be as high or higher than that of locomotion alone (Priede, 1985). Model code can be found at https://github.com/Philipp-Neubauer/AdaptiveActivityModel; an interactive version of the model can be found here: https://dragonfly-science.shinyapps.io/SizingtheFxofClimateChange/.

## Results

**Figure 3:**
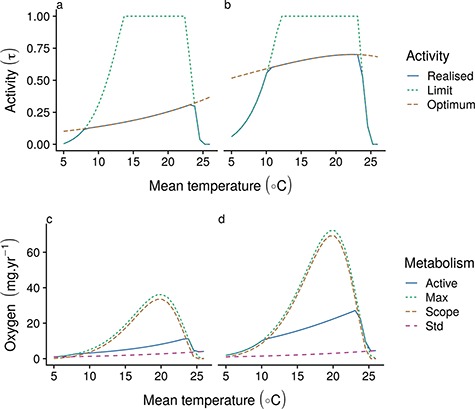
Optimum (red long-dashed), maximum (green short-dashed) and realized (blue solid lines) activity levels (top row [a,b]) at increasing temperatures for a 10 g fish with a dome-shaped MOS with increasing temperature (maximum at 20°C), with corresponding oxygen demand (bottom row [c,d]); MOS (green short-dashed), standard metabolism (red long-dashed) and realized (active; blue solid lines) metabolic demand, as well as metabolic scope (orange long-dashed) at activity level τ, for slow life history (a and c) and fast life history (b and d).

**Figure 4:**
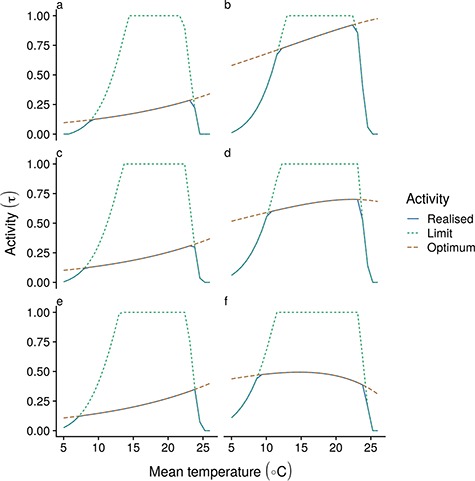
Optimum (red long-dashed), maximum (green short-dashed) and realized (blue solid lines) activity levels (left column) at increasing temperatures through ontogeny for a for fish with a cm at 1.25 g (5 cm; a–b); 10 g (10 cm; c–d) and 80 g (20 cm; e–f), for slow strategy (slow life history; left column [a,c,e]) and fast strategy (fast life history; right column [b,d,f]) species with a dome-shaped MMR with respect to temperature.

Increasing metabolic demands at higher temperatures leads to increased activity levels in order to optimize energy gains relative to mortality risk (Fig. 3). This difference in activity level is especially pronounced in slow strategy species, for which the overall activity level is markedly lower and which consistently show higher activity over all sizes for the simulated life history (Fig. 4). A similarly higher activity level is observed for small fast strategy individuals (e.g. post-larval) for which even initial activity levels are very high (Fig. 4). For these individuals, the higher activity levels and metabolic demands lead to an active metabolic rate that is close to their MMR. For all other sizes across the two trait scenarios, oxygen is only limiting to activity at the extremes of the simulated temperature range (Fig. 3) and only for species with a dome-shaped MOS with respect to temperature. For species with a rising MOS with temperature, oxygen is not limiting (Fig. S2). However, larger fast strategy individuals are predicted to show a slightly dome-shaped relationship between activity levels and temperature at intermediate sizes, and slightly decreasing activity levels in response to temperature at large sizes, despite available aerobic scope for activity. This adjustment is a function of metabolic activity costs assumed here—if we assume smaller metabolic costs for activity, activity levels are always higher at higher temperature (Fig. S3).

**Figure 5:**
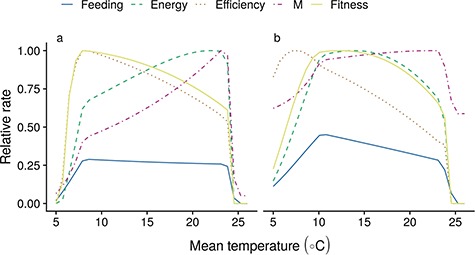
Feeding level (blue solid line), available energy *P* (green long-dashed), efficiency (orange dotted), mortality (red dotted-dashed) and the ratio of *P* to *M* (yellow solid) are impacted by changing activity and the metabolic response to temperature for a growing fish (cm) at 10 cm length (10 g). Responses are shown for slow and fast strategy (a and b, respectively), for species with and without oxygen limitation (left and right columns, respectively). Energy, mortality and fitness are plotted relative to their maximum over all temperatures.

Temperature and metabolic demand-driven adjustments to the activity level lead to substantial changes in performance-related metrics in both trait scenarios (Fig. 5). For slow strategy species, higher activity levels at warmer temperatures lead to relatively stable feeding levels, but a substantially higher mortality coupled with slightly increased available energy leads to an overall decline in the ratio of *P* to *M*. Available energy shows a dome-shaped response to temperature in slow strategists and is maximized at relatively high temperatures. However, it is limited by oxygen availability only at high temperatures in species with a dome-shaped MOS. Production efficiency follows a near opposite trend due to the relatively flat response in the feeding level *f*, but temperature-driven increases in maximum consumption.

For fast strategists, the relatively modest response in activity levels at all but the smallest sizes leads to a decline in feeding levels, which causes a largely dome-shaped response of available energy and growth efficiency to warmer temperatures (Fig. 5). Again, production efficiency peaks at relatively low temperatures, but for fast life histories, available energy *P* peaks at much lower temperatures. Given the relatively flat mortality levels, the ratio of *P*/*M* largely follows the trend in *P*.

**Figure 6:**
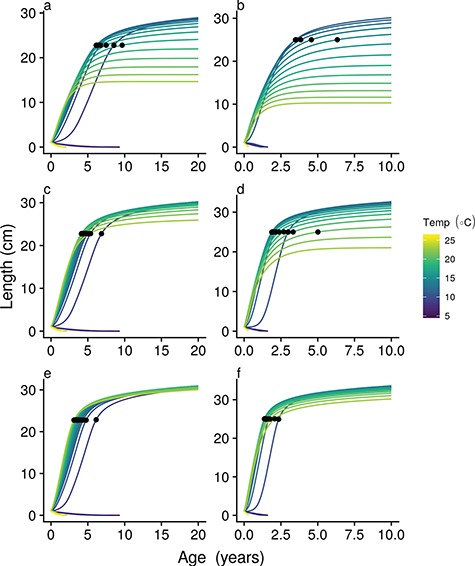
On ecological timescales, increasing temperature (purple to yellow growth curves) modifies growth, and maturation age changes according to the reaction norm (black dots at 50% allocation to reproduction), whereas asymptotic size is affected by changes in absolute energy available for growth. Growth curves are shown for slow (left column [a,c,e]) and fast strategists (right column [b,d,f]), at increasing food availability from top (a/b) to bottom (e/f). Baseline resource availability assumed in all other simulations is that shown in panels c and d.

Simulated growth curves illustrate the ontogenetic consequences of higher temperatures (Fig. 6). For both trait scenarios, fastest growth occurred at relatively high temperatures, with declining growth for oxygen limited species at the highest temperatures (Fig. S4). This can be explained by ontogenetic shifts in temperature optima for growth (Fig. S5); for small individuals, available energy and growth consistently peak at high temperatures, but this peak rapidly moves to lower temperatures as individuals in either trait scenario grow. For large individuals, growth is optimized at relatively low temperatures, leading to larger asymptotic size at lower temperatures.

**Figure 7:**
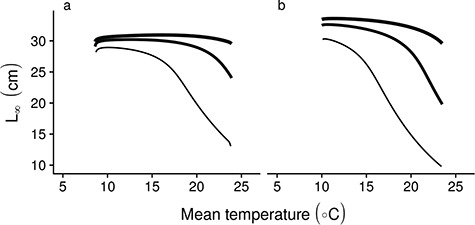
Interactive effects of food availability and temperature of the asymptotic size (${L}_{\infty }$), with increasing line width showing increasing food resource availability for (a) slow and (b) fast life-history species.

Resource availability strongly modulates this growth response to temperature; low-resource availability leads to strong differences in asymptotic size, whereas high food availability leads to fast growth and larger asymptotic length at high temperatures (Figs 6 and 7). In addition, in very resource poor conditions, individuals may not grow to reproductive size in our scenario of a flat MRN. Overall growth responses to temperature are not strongly affected by the assumed slope of the MRN (Figs S6 and S7), although a negatively sloped MRN does ensure maturation in low-resource environments.

**Figure 8:**
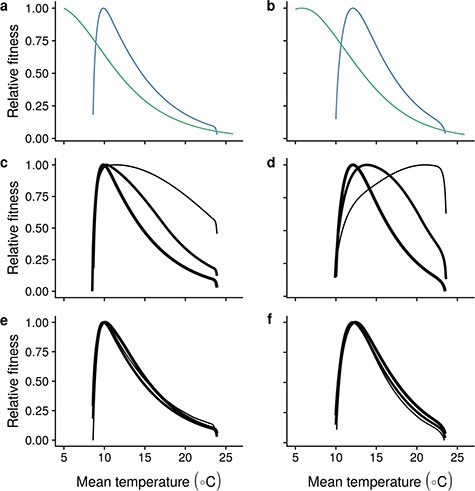
Fitness (${R}_0$), relative to maximum fitness within (a and b) oxygen limited (blue) and non-oxygen limited (teal) for slow (left column) and fast strategy (right column) species at the assumed MRN and default parameters, and (c and d) changes in relative fitness with respect to temperature resulting from decreasing levels of activity cost symbolized by decreasing line width, and (e and f) changes in relative fitness with respect to temperature as a function of increasing food availability (increasing line width).

Overall fitness consequences mirror trends in the ratio of *P*/*M* (Fig. 8a), which can be seen as a short-sighted approximation to overall fitness optimization (Sainmont *et al*., 2015). With increasing temperatures, fitness declines at our basic parameter settings, in opposition to growth and aerobic scope. At low temperatures, fitness is limited by aerobic scope, with the magnitude determined by the extend of doming in aerobic scope. Note that this limitation through the aerobic scope appears at higher temperatures than apparent from Fig. 3, reflecting stronger limitation of oxygen on growth during early life (Fig. S5). Fitness trends with temperature are strongly dependent on metabolic costs of activity (Fig. 8b), and changing the activity cost to lower values attenuates the decline in fitness with temperature for slow strategy species and moves the fitness optimum to higher temperatures for fast strategy species. Similarly, increased food availability can lead to a slower decline of fitness with temperature, especially for fast strategists (Fig. 8c).

## Discussion

In this study, we attempt to provide a general mechanistic basis for exploring thermal sensitivities of ectotherm organisms. Much of the recent debate about the validity of projected climate change impact on ectotherms, and fish in particular, has revolved around the validity of particular concepts, such as the OCLTT and projections based on the gill-oxygen limitation theory (Pauly and Cheung, 2017; Lefevre *et al*., 2018). We attempted to go beyond this debate by developing a model that allows for general insights about the temperature response in ectotherms, while being specific enough to mechanistically articulate aspects of physiology and ecology that are fundamental to organism response to temperature. The general model and its parameter values are also easily adjusted to reflect particular organisms or theories.

It has been argued that the OCLTT as a concept provides a basis to explain observed responses to climate change on the basis of oxygen limitation via the aerobic scope (Pörtner and Farrell, 2008; Pörtner, 2010), and simple oxygen budgets have been used to predict metabolic constraints on organismal activity due to warming ocean temperatures (Deutsch *et al*., 2015). As a conceptual framework, however, the OCLTT is subject not only to semantic dispute but also criticism of its core concept of oxygen limitation (Lefevre, 2016; Jutfelt *et al*., 2018).

Our quantitative thermal impact model generalizes existing ecophysiological models for particular species and stocks (Hufnagl and Peck, 2011; Holt and Jorgensen, 2014, 2015) and allows to develop a more nuanced understanding of interactions between temperature, oxygen limitation and ecology for species with varying traits. In line with the conceptual framework of Fry’s aerobic scope and the OCLTT, our model suggests that oxygen limitation can be a potentially important ecological driver, especially at extreme temperatures for species with declining MOSs in such temperature regimes. At the onset of this limitation, ecological parameters change drastically, and both growth and mortality are strongly impacted. This limitation closely mimics limitations seen in wild fish (Myrick and Cech, 2000) and is in line with observations that fish often seek specific water temperatures to optimize metabolic function (Claireaux *et al*., 1995; Armstrong *et al*., 2013). Fitness, however, appears to be limited through the metabolic scope primarily via limitations at temperature extremes and impact on particular life-history stages. For instance, oxygen limitation is a more severe constraint for small individuals (Fig. S5) and thereby can limit growth performance early in life, impacting overall fitness. Furthermore, changes in environmental oxygen supply, if beyond an organism’s ability to compensate via passive or active compensation mechanisms, will induce an overall lower aerobic scope and lead to an earlier onset of oxygen limitation, but such scenarios do not change the qualitative predictions from our model.

Variations in performance metrics away from temperature extremes are primarily affected by the interaction of temperature-driven metabolic demands with optimal feeding behaviour. Predictions from our model, in line with metabolic experiments and species-specific physiological predictions (Holt and Jorgensen, 2014), suggest that routine activity, including normal swimming behaviour, feeding and digestion, usually lead to routine metabolic rates that are well below the MOS, even in fish with high metabolism (Priede, 1985; Lucas and Priede, 1992). Strenuous swimming activity, for example, usually only makes up a small proportion of the standard energy budget in fish (Priede 1977, 1985). Furthermore, as a limit for long-term performance, the MOS does not usually impose a limitation on short-term energy demands, as fish can incur oxygen debt during swimming bursts during which the MOS is exceeded (Brett, 1972; Priede, 1985). A logical conclusion is that the metabolic scope is only limiting to performance at extreme temperatures where MOS is low due to impaired oxygen delivery.

In many species, both the aerobic scope and growth peak at relatively high temperatures within the potential thermal range, yet species are often found at temperatures lower than these optima (Magnuson and DeStasio, 1997; Claireaux *et al*., 2000). Previous explanations of this niche occupation paradox involved environmental factors that narrow the thermal niche or behaviour that optimizes thermal performance across available habitats (Magnuson and DeStasio, 1997; Claireaux *et al*., 2000; Martin and Huey, 2008). In nearly all our simulation scenarios, fitness is predicted to decline with increasing temperature, and our model therefore provides a complementary explanation to those based on behavioural thermoregulation in variable environments (Martin and Huey, 2008). This decline with temperature also leads to a parsimonious explanation for the relationship between growth performance and fitness at varying temperatures.

Resource availability imposes a strong environmental constraint on organisms, with all aspects from optimal activity levels, mortality and available energy for growth ultimately influenced by available food resources. Changes in food resource availability thus influence individual temperature response directly via available energy, and indirectly, through altered energetic requirements to procure food and changes in mortality due to changes in the optimal activity level. In low-food environments, changing energetic demands with temperature are not easily adjusted for, and any adjustment demands higher energetic costs and mortality risk. This environment-driven change in costs of temperature adjustments leads to the strong modulation of the growth response, as well as changing gradients in fitness with temperature in these environments. In particular, in low-food environments, asymptotic size is strongly reduced at high temperatures, whereas this is not necessarily the case in high-food environments.

Our model prediction of declining fitness with temperature is particularly sensitive to activity costs, with low activity costs leading to increased fitness at warmer temperatures. Our default parametrization is based on the assumption that activity cost reflects the cost of maximal activity (i.e. cost of τ = 1) and has the same pay-off for all life histories. This approximation may not reflect actual activity cost as swimming at MMR in fast predatory fish is far more efficient than swimming of sluggish fish, such as flatfish (Priede, 1985). Such an efficiency gain may occur as a result of more efficient form or physiology, or simply by reduced drag at large size (e.g., whale sharks), and may be a key requirement to the viability of active pelagic predatory fish in tropical waters.

Optimal activity is predicted to be higher at warmer temperature in nearly all cases, but this finding is sensitive to the cost of activity—at higher cost relative to the potential pay-off, activity may even decline at high temperatures. Predictions of increased activity are supported by many observations in experimental and field conditions for both larval and adult fish (Brown *et al*., 1989; Claireaux *et al*., 1995; Biro *et al*., 2007; Sswat *et al*., 2018). This increase of activity often occurs despite increased mortality (Biro *et al*., 2007; Sswat *et al*., 2018) and serves the necessity to offset increased metabolic expenses. Only large individuals of the simulated fast strategy species will optimally decrease activity as a result of increased temperature. In this case, additional activity will lead to comparatively small gains from feeding relative to the cost of activity and SDA, owing to the non-linearity of the functional response.

Taken together, the physiological processes and optimization described in our model provide a mechanistic underpinning for observations about changes of ecological rates, such as increasing or dome-shaped consumption or attack rates with temperature (Biro *et al*., 2007; Englund *et al*., 2011; Rall *et al*., 2012). Depending on the strength of oxygen limitation and the development of the optimal activity level over the range of temperatures considered, attack rates and feeding rates may appear to be steadily increasing via increased optimal activity, or dome-shaped from oxygen limitation or dome-shaped optimal activity. Thus, rather than assuming *ad hoc* changes in ecological rates in response to temperature that may not be transferable between species and traits, the change in these rates may be mechanistically described in terms of optimal ecological adjustments to physiological constraints.

Similarly, our model provides a mechanistic basis for the temperature-size rule in ectotherms (Atkinson, 1994), without needing to evoke direct changes in ecological rates with temperature. The physiological basis leads to heterogeneous predictions about growth trajectories over ranges of temperature, with various degrees of nesting (i.e. non-crossing) and crossing of growth trajectories possible depending on ecological conditions (e.g. food availability) and physiological traits (Fig. 6; Fig. S4). Due to its reliance on physiological traits and their interaction with ecological variables, our model provides a multivariate framework to predict heterogeneous temperature impacts on size and growth (Angilletta *et al*., 2004).

In order to provide a general framework, our model set-up is deliberately minimalist, and probably under-parametrized to reflect ecological and life-history aspects of particular species, such as migrations, social behaviour or seasonal energy requirements. As such, this framework provides a null model to assess the diversity of possible responses in fish, and other ectotherms, to temperature in a highly simplified system. Nevertheless, it provides a starting point from which to explore the importance of costs and benefits of particular life histories and thermal adaptations. For instance, a recent species-specific ecophysiological model for cod (*Gadus morhua*) that includes similar physiological constraints to our model predicted relatively high fitness at high temperatures (Holt and Jorgensen, 2014, 2015). Although this difference is potentially due to the different activity cost coefficients, foraging assumptions and species-specific parametrizations in their model, differences may also be due to key assumptions about optimal reproduction; the model of Holt and Jorgensen (2014) assumed that reproductive investment is instantaneously optimized in a changing climate, pointing to the possibility that adaptation of reproductive strategies could offset potential fitness declines with increasing temperatures.

## Conclusion

The importance of the interaction between ecology, bioenergetics and oxygen limitations in deriving realistic predictions about temperature impacts on ecological rates and fitness calls into question predictions for climate change impacts based on simple models of growth alone (Cheung *et al*., 2013; Pauly and Cheung, 2017). We suggest that the general trait-based approach presented here provides a parsimonious compromise between simplistic approximations that may provide misleading predictions about future ecosystems (Brander *et al*., 2013; Lefevre *et al*., 2017) and more complex ecophysiological models such as dynamic energy budget models (Guiet *et al*., 2016) and species-specific ecophysiology models (Hufnagl and Peck, 2011; Holt and Jorgensen, 2014, 2015). In addition, our model provides a more explicit, physiology-based mechanistic model to derive general predictions about temperature effects on ectotherms than previous general frameworks such as the OCLTT. Predictions from the OCLTT are both contributing to patterns in fitness and ecological rates shown here, but are also only part of the picture, and we suggest that future improvements of predictive frameworks should center on model criticism and improvements and leave behind semantic discussions about conceptual constructs that are difficult to explicitly link to data. Improved ecophysiological models will provide a more robust basis for incorporating ecophysiology into tactical management and strategic conservation planning (McKenzie *et al*., 2016; Patterson *et al*., 2016).

## Supplementary Material

FigureS1

FigureS2

FigureS3

FigureS4

FigureS5

FigureS6

FigureS7

## Appendix 1: MRN

A flat MRN leads to a logistic allocation to reproduction that is only dependent on size and independent of age (Fig. S1).

To express an MRN that varies as a function of age, we can reparametrize the MRN as

(12) \begin{equation*} \phi (z)=1/\left(1+\exp \left(-(cz)\right)\right), \textrm{where} \end{equation*}

(13) \begin{equation*} z\left(t,{w}(t)\right)=\left({w}(t)-{w}^{\ast}\right)\cos \left(a\tan (b)\right)-t \sin \left(a\tan (b)\right) \end{equation*}

rotates the coordinate system about the slope *b* of the reaction norm, *t* is the age, *w* is the mass, ${w}*^*$ is the intercept of the reaction norm and *c* determines how rapidly energy allocation shifts from somatic growth to reproduction.

## References

[ref1] Andersen KH, Farnsworth KD, Pedersen M, Gislason H, Beyer JE (2009) How community ecology links natural mortality, growth, and production of fish populations. ICES J Mar Sci 66: 1978–1984.

[ref2] Andersen KH, Jacobsen NS, Jansen T, Beyer JE (2017) When in life does density dependence occur in fish populations? Fish Fish 18: 656–667.

[ref3] Angilletta MJ, Steury TD, Sears MW (2004) Temperature, growth rate, and body size in ectotherms: fitting pieces of a life-history puzzle. Integr Comp Biol 44: 498–509. 21676736 10.1093/icb/44.6.498

[ref4] Armstrong JB, Schindler DE, Ruff CP, Brooks GT, Bentley KE, Torgersen CE (2013) Diel horizontal migration in streams: juvenile fish exploit spatial heterogeneity in thermal and trophic resources. Ecology 94: 2066–2075. 24279277 10.1890/12-1200.1

[ref5] Atkinson D (1994) Temperature and organism size: a biological law for ectotherms? Adv Ecol Res 25: 1–1.

[ref6] Biro PA, Post JR, Booth DJ (2007) Mechanisms for climate-induced mortality of fish populations in whole-lake experiments. Proc Natl Acad Sci U S A 104: 9715–9719. 17535908 10.1073/pnas.0701638104 PMC1887605

[ref7] Brander K, Neuheimer A, Andersen KH, Hartvig M (2013) Overconfidence in model projections. ICES J Mar Sci 70: 1065–1068.

[ref8] Brett J (1965) The relation of size to rate of oxygen consumption and sustained swimming speed of sockeye salmon (oncorhynchus nerka). J Fish Res Board Can 22: 1491–1501.

[ref9] Brett JR (1972) The metabolic demand for oxygen in fish, particularly salmonids, and a comparison with other vertebrates. Respir Physiol 14: 151–170. 5042150 10.1016/0034-5687(72)90025-4

[ref10] Brown JA, Pepin P, Methven DA, Somerton DC (1989) The feeding, growth and behaviour of juvenile cod, gadus morhua l., in cold environments. J Fish Biol 35: 373–380.

[ref11] Brown JH, Gillooly JF, Allen AP, Savage VM, West GB (2004) Toward a metabolic theory of ecology. Ecology 85: 1771–1789.

[ref12] Charnov EL, Gislason H, Pope JG (2013) Evolutionary assembly rules for fish life histories. Fish Fish 14: 213–224.

[ref13] Cheung WW, Sarmiento JL, Dunne J, Frölicher TL, Lam VW, Palomares MD, Watson R, Pauly D (2013) Shrinking of fishes exacerbates impacts of global ocean changes on marine ecosystems. Nat Clim Chang 3: 254–258.

[ref14] Claireaux G, Lefrançois C (2007) Linking environmental variability and fish performance: integration through the concept of scope for activity. Philos Trans R Soc Lond B Biol Sci 362: 2031–2041. 17472923 10.1098/rstb.2007.2099 PMC2442852

[ref15] Claireaux G, Webber D, Kerr S, Boutilier R (1995) Physiology and behaviour of free-swimming Atlantic cod (gadus morhua) facing fluctuating temperature conditions. J Exp Biol 198: 49–60. 9317317 10.1242/jeb.198.1.49

[ref16] Claireaux G, Webber DM, Lagardère J-P, Kerr SR (2000) Influence of water temperature and oxygenation on the aerobic metabolic scope of Atlantic cod (gadus morhua). J Sea Res 44: 257–265.

[ref17] Deutsch C, Ferrel A, Seibel B, Pörtner H-O, Huey RB (2015) Climate change tightens a metabolic constraint on marine habitats. Science 348: 1132–1135. 26045435 10.1126/science.aaa1605

[ref18] Dieckmann U, Heino M (2007) Probabilistic maturation reaction norms: their history, strengths, and limitations. Mar Ecol Prog Ser 335: 253–269.

[ref19] Englund G, Ohlund G, Hein CL, Diehl S (2011) Temperature dependence of the functional response. Ecol Lett 14: 914–921. 21752171 10.1111/j.1461-0248.2011.01661.x

[ref20] FEJ Fry (1947) Effects of the Environment on Animal Activity. Publ. Out. Fish. Res. Lab., 55: 1–62.

[ref21] Gilliam JF, Fraser DF (1987) Habitat selection under predation hazard: test of a model with foraging minnows. Ecology 68: 1856–1862. 29357169 10.2307/1939877

[ref22] Gillooly JF, Brown JH, West GB, Savage VM, Charnov EL (2001) Effects of size and temperature on metabolic rate. Science 293: 2248–2251. 11567137 10.1126/science.1061967

[ref23] Glazier DS (2009) Activity affects intraspecific body-size scaling of metabolic rate in ectothermic animals. J Comp Physiol B 179: 821–828. 19387653 10.1007/s00360-009-0363-3

[ref24] Gnauck A, Straškraba M (2013) Freshwater Ecosystems: Modelling and Simulation Vol 8 Elsevier, Amsterdam, Netherlands.

[ref25] Guiet J, Aumont O, Poggiale J-C, Maury O (2016) Effects of lower trophic level biomass and water temperature on fish communities: a modelling study. Prog Oceanogr 146: 22–37.

[ref26] Hartvig M, Andersen KH, Beyer JE (2011) Food web framework for size-structured populations. J Theor Biol 272: 113–122. 21146543 10.1016/j.jtbi.2010.12.006

[ref27] Holt RE, Jorgensen C (2014) Climate warming causes life-history evolution in a model for Atlantic cod (gadus morhua). Conserv Physiol 2: doi: 10.1093/conphys/cou050. PMC4806736 27293671

[ref28] Holt RE, Jorgensen C (2015) Climate change in fish: effects of respiratory constraints on optimal life history and behaviour. Biol Lett 11: 20141032–20141032. 25673000 10.1098/rsbl.2014.1032 PMC4360111

[ref29] Hufnagl M, Peck MA (2011) Physiological individual-based modelling of larval Atlantic herring (clupea harengus) foraging and growth: insights on climate-driven life-history scheduling. ICES J Mar Sci 68: 1170–1188

[ref30] Iftikar FI, Hickey AJR (2013) Do mitochondria limit hot fish hearts? Understanding the role of mitochondrial function with heat stress in notolabrus celidotus. PLoS One 8: e64120. 23724026 10.1371/journal.pone.0064120 PMC3665896

[ref31] Jeschke JM, Kopp M, Tollrian R (2002) Predator functional responses: discriminating between handling and digesting prey. Ecol Monogr 72: 95–112.

[ref32] Jutfelt F, Norin T, Ern R, Overgaard J, Wang T, McKenzie DJ, Lefevre S, Nilsson GE, Metcalfe NB, Hickey AJR (2018) Oxygen-and capacity-limited thermal tolerance: blurring ecology and physiology. J Exp Biol 221: jeb169615. 29321291 10.1242/jeb.169615

[ref33] Killen SS, Atkinson D, Glazier DS (2010) The intraspecific scaling of metabolic rate with body mass in fishes depends on lifestyle and temperature. Ecol Lett 13: 184–193. 20059525 10.1111/j.1461-0248.2009.01415.x

[ref34] Kozlowski J, Czarnoleski M, Danko M (2004) Can optimal resource allocation models explain why ectotherms grow larger in cold? Integr Comp Biol 44: 480–493. 21676734 10.1093/icb/44.6.480

[ref35] Lefevre S (2016) Are global warming and ocean acidification conspiring against marine ectotherms? A meta-analysis of the respiratory effects of elevated temperature, high CO2 and their interaction. Conserv Physiol 4: doi: 10.1093/conphys/cow009. PMC4922249 27382472

[ref36] Lefevre S, McKenzie DJ, Nilsson GE (2017) Models projecting the fate of fish populations under climate change need to be based on valid physiological mechanisms. Glob Chang Biol 23, 3449–3459. 28168760 10.1111/gcb.13652

[ref37] Lefevre S, McKenzie DJ, Nilsson GE (2018) In modelling effects of global warming, invalid assumptions lead to unrealistic projections. Glob Chang Biol 24: 553–556. 29120513 10.1111/gcb.13978

[ref38] Lefrancois C, Claireaux G (2003) Influence of ambient oxygenation and temperature on metabolic scope and scope for heart rate in the common sole solea solea. Mar Ecol Prog Ser 259: 273–284.

[ref39] Lucas MC, Priede IG (1992) Utilization of metabolic scope in relation to feeding and activity by individual and grouped zebrafish, brachydanio rerio (Hamilton-Buchanan). J Fish Biol 41: 175–190.

[ref40] Lorenzen K, Camp EV (2018) Density-dependence in the life history of fishes: when is a fish recruited? Fish Resin press 10.1016/j.fishres.2018.09.024.

[ref41] Magnuson JJ, BT DeStasio (1997) Thermal niche of fishes and global warming. In Wood C, McDonald D, eds, Global Warming: Implications for Freshwater and Marine Fish, Vol 61 Cambridge University Press, Cambridge, UK, pp 377–408.

[ref42] Martin TL, Huey RB (2008) Why “suboptimal” is optimal: Jensen’s inequality and ectotherm thermal preferences. Am Nat 171: 102–118. 10.1086/527502 18271721

[ref43] Marty L, Dieckmann U, Rochet M-J, Ernande B (2011) Impact of environmental covariation in growth and mortality on evolving maturation reaction norms. Am Nat 177: E98–E118. 21460562 10.1086/658988

[ref44] McKenzie DJ Axelsson M, Chabot D, Claireaux G, Cooke SJ, Corner RA, De Boeck G, Domenici P, Guerreiro PM, Hamer B et al. (2016) Conservation physiology of marine fishes: state of the art and prospects for policy. Conserv Physiol 4: doi: 10.1093/conphys/cow046. PMC5070530 27766156

[ref45] Moffett ER, Fryxell DC, Palkovacs EP, Kinnison MT, Simon KS (2018) Local adaptation reduces the metabolic cost of environmental warming. Ecology 99: 2318–2326. 30030930 10.1002/ecy.2463

[ref46] Myrick CA, Cech JJ (2000) Temperature influences on California rainbow trout physiological performance. Fish Physiol Biochem 22: 245–254.

[ref47] Parmesan C, Yohe G (2003) A globally coherent fingerprint of climate change impacts across natural systems. Nature 421: 37–42. 12511946 10.1038/nature01286

[ref48] Patterson DA, Cooke SJ, Hinch SG, Robinson KA, Young N, Farrell AP, Miller KM (2016) A perspective on physiological studies supporting the provision of scientific advice for the management of Fraser River sockeye salmon ( *Oncorhynchus nerka*). Conserv Physiol 4: doi: 10.1093/conphys/cow026. PMC5001150 27928508

[ref49] Pauly D, Cheung WWL (2017) Sound physiological knowledge and principles in modeling shrinking of fishes under climate change. Glob Chang Biol 24: e15–e26. 28833977 10.1111/gcb.13831

[ref50] Pinsky ML, Worm B, Fogarty MJ, Sarmiento JL, Levin SA (2013) Marine taxa track local climate velocities. Science 341: 1239–1242. 24031017 10.1126/science.1239352

[ref51] Priede IG (1977) Natural selection for energetic efficiency and the relationship between activity level and mortality. Nature 267: 610–611. 876379 10.1038/267610a0

[ref52] Priede IG (1985) Metabolic scope in fishes. In Fish Energetics. Springer, Dordrecht, pp 33–64.

[ref53] Pörtner H-O (2010) Oxygen-and capacity-limitation of thermal tolerance: a matrix for integrating climate-related stressor effects in marine ecosystems. J Exp Biol 213: 881–893. 20190113 10.1242/jeb.037523

[ref54] Pörtner HO, Farrell AP (2008) Physiology and climate change. Science 322: 690–692. 18974339 10.1126/science.1163156

[ref55] Rall BC, Brose U, Hartvig M, Kalinkat G, Schwarzmüller F, Vucic-Pestic O, Petchey OL (2012) Universal temperature and body-mass scaling of feeding rates. Philos Trans R Soc Lond B Biol Sci 367: 2923–2934. 23007080 10.1098/rstb.2012.0242 PMC3479751

[ref56] Sainmont J, Andersen KH, Thygesen UH, Fiksen O, Visser AW (2015) An effective algorithm for approximating adaptive behavior in seasonal environments. Ecol Model 311: 20–30.

[ref57] Salin K, Auer SK, Anderson GJ, Selman C, Metcalfe NB (2016) Inadequate food intake at high temperatures is related to depressed mitochondrial respiratory capacity. J Exp Biol 219: 1356–1362. 26944497 10.1242/jeb.133025

[ref58] Sandblom E, Clark TD, Gräns A, Ekström A, Brijs J, Sundström LF, Odelström A, Adill A, Aho T, Jutfelt F (2016) Physiological constraints to climate warming in fish follow principles of plastic floors and concrete ceilings. Nat Commun 7: 11447. 27186890 10.1038/ncomms11447 PMC4873662

[ref59] Sentis A, Hemptinne J-L, Brodeur J (2013) Parsing handling time into its components: implications for responses to a temperature gradient. Ecology 94: 1675–1680. 24015511 10.1890/12-2107.1

[ref60] Sswat M, Stiasny MH, Jutfelt F, Riebesell U, Clemmesen C (2018) Growth performance and survival of larval Atlantic herring, under the combined effects of elevated temperatures and CO2. PLoS One 13: e0191947. 29370273 10.1371/journal.pone.0191947 PMC5785030

[ref61] Stuart-Smith RD, Edgar GJ, Barrett NS, Kininmonth SJ, Bates AE (2015) Thermal biases and vulnerability to warming in the world’s marine fauna. Nature 528: 88–92. 26560025 10.1038/nature16144

[ref62] Verberk WC, Bilton DT, Calosi P, Spicer JI (2011) Oxygen supply in aquatic ectotherms: partial pressure and solubility together explain biodiversity and size patterns. Ecology 92: 1565–1572. 21905423 10.1890/10-2369.1

[ref63] Verberk WCEP, Overgaard J, Ern R, Bayley M, Wang T, Boardman L, Terblanche JS (2016) Does oxygen limit thermal tolerance in arthropods? A critical review of current evidence. Comp Biochem Physiol A Mol Integr Physiol 192: 64–78. 26506130 10.1016/j.cbpa.2015.10.020 PMC4717866

[ref64] Vucic-Pestic O, Ehnes RB, Rall BC, Brose U (2011) Warming up the system: higher predator feeding rates but lower energetic efficiencies. Glob Chang Biol 17: 1301–1310.

[ref65] Walther G-R, Post E, Convey P, Menzel A, Parmesan C, Beebee TJC, Fromentin J-M, Hoegh-Guldberg O, Bairlein F (2002) Ecological responses to recent climate change. Nature 416: 389–395. 11919621 10.1038/416389a

