# Supplementary figures and images for: Thermal performance of fish is explained by an interplay between physiology, behaviour and ecology
Source: Conserv Physiol. 2019 Jun 10;7(1):coz025. doi: 10.1093/conphys/coz025 (PMC6659025; doi:10.1093/conphys/coz025)

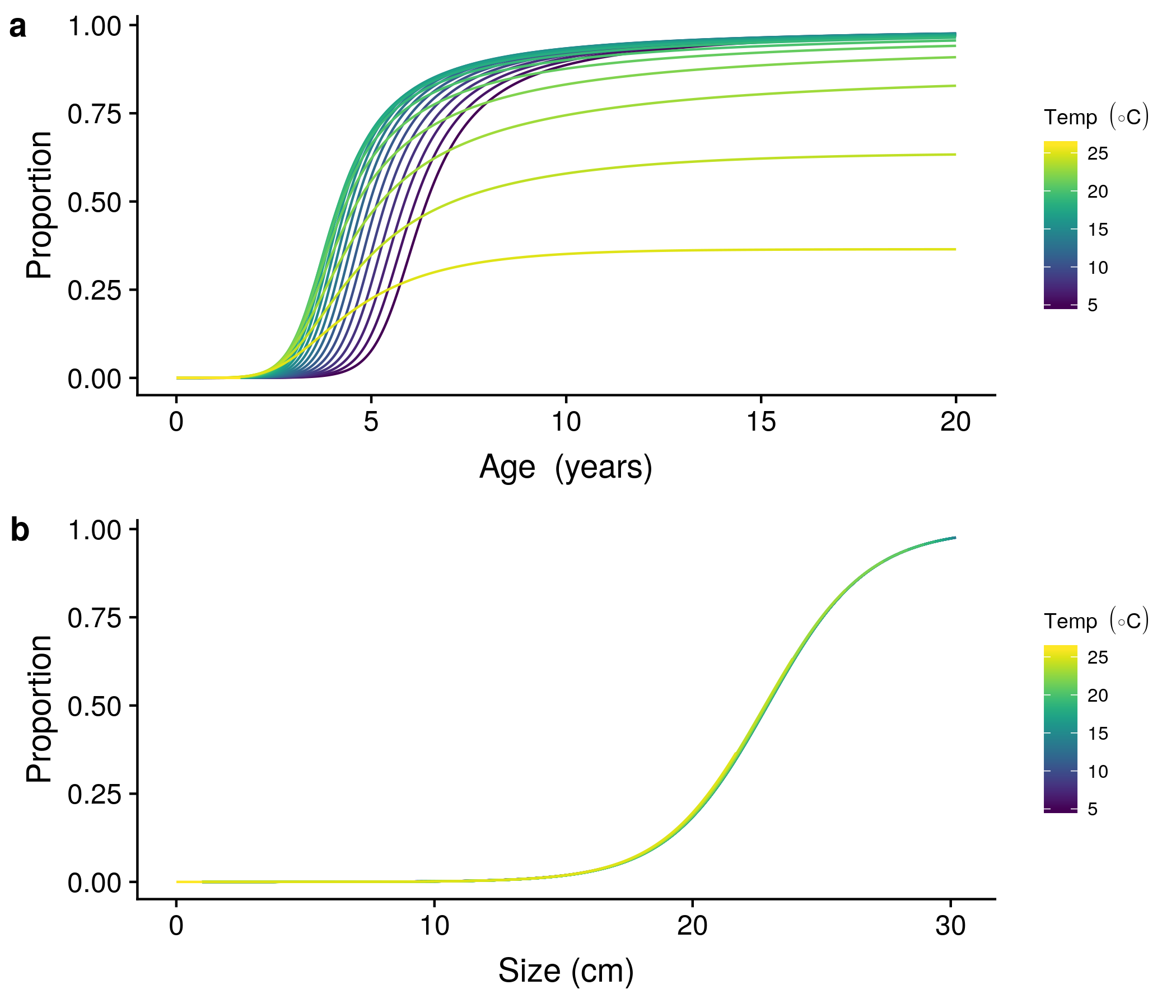

Supplement: FigureS1 [file figures1.png]

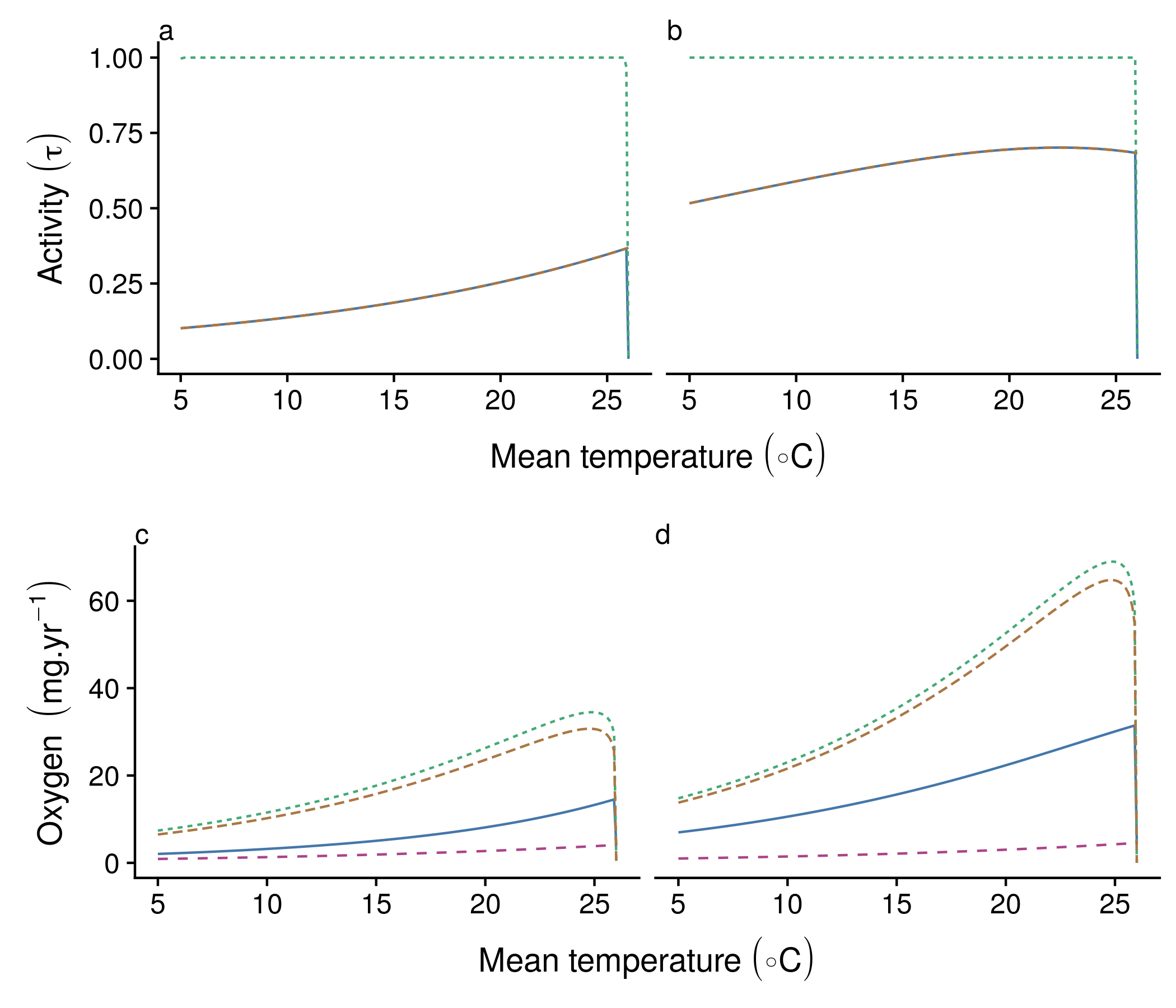

Supplement: FigureS2 [file figures2.png]

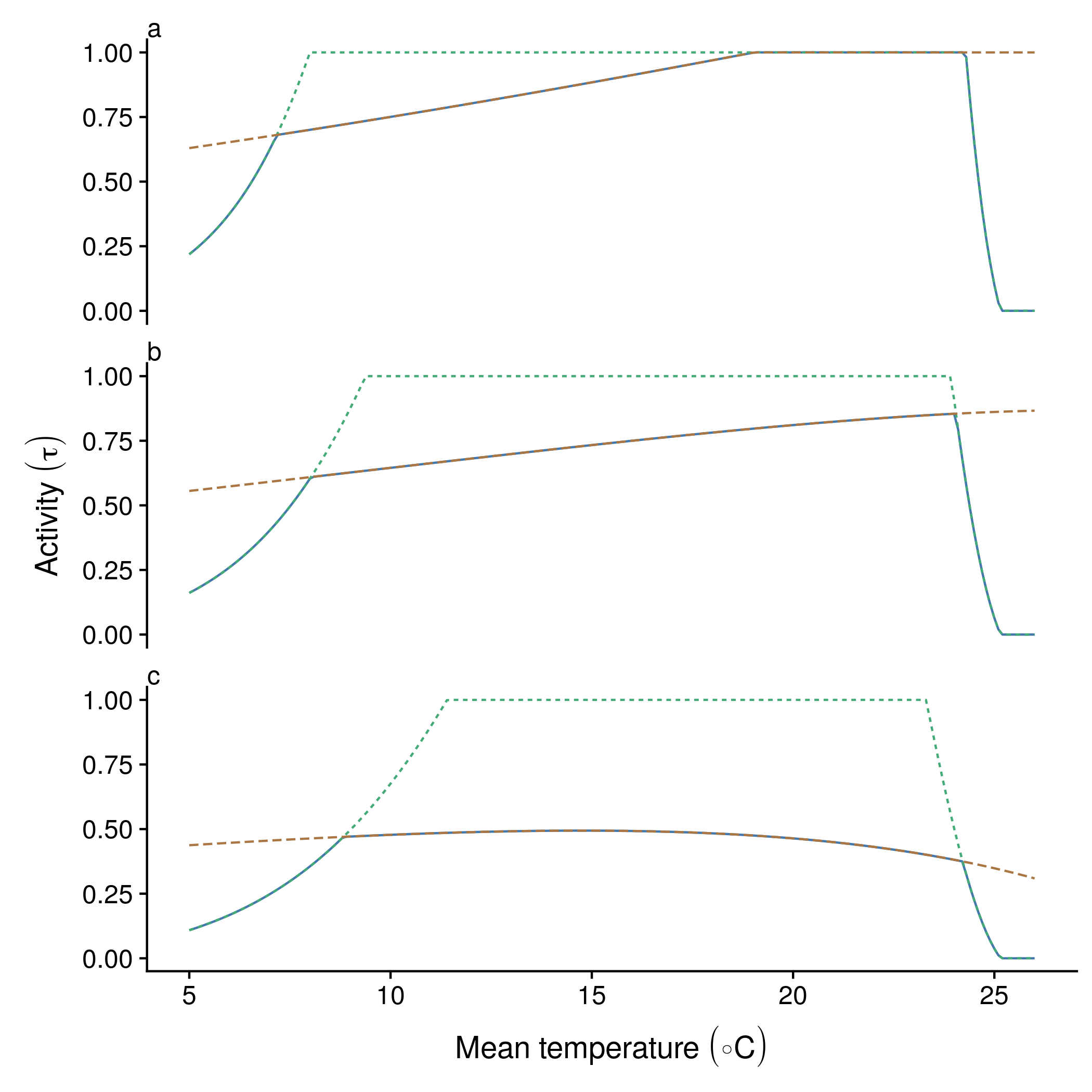

Supplement: FigureS3 [file figures3.png]

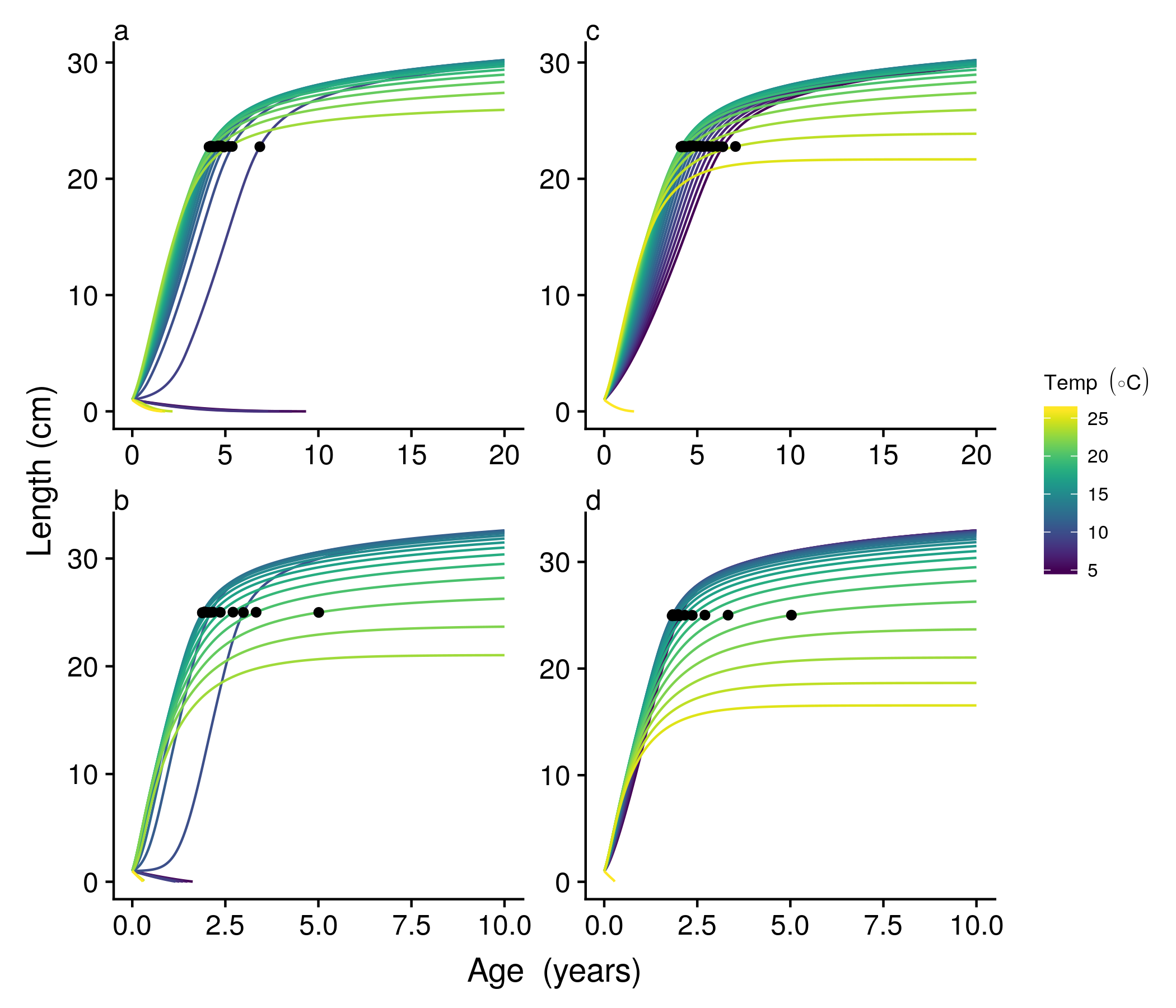

Supplement: FigureS4 [file figures4.png]

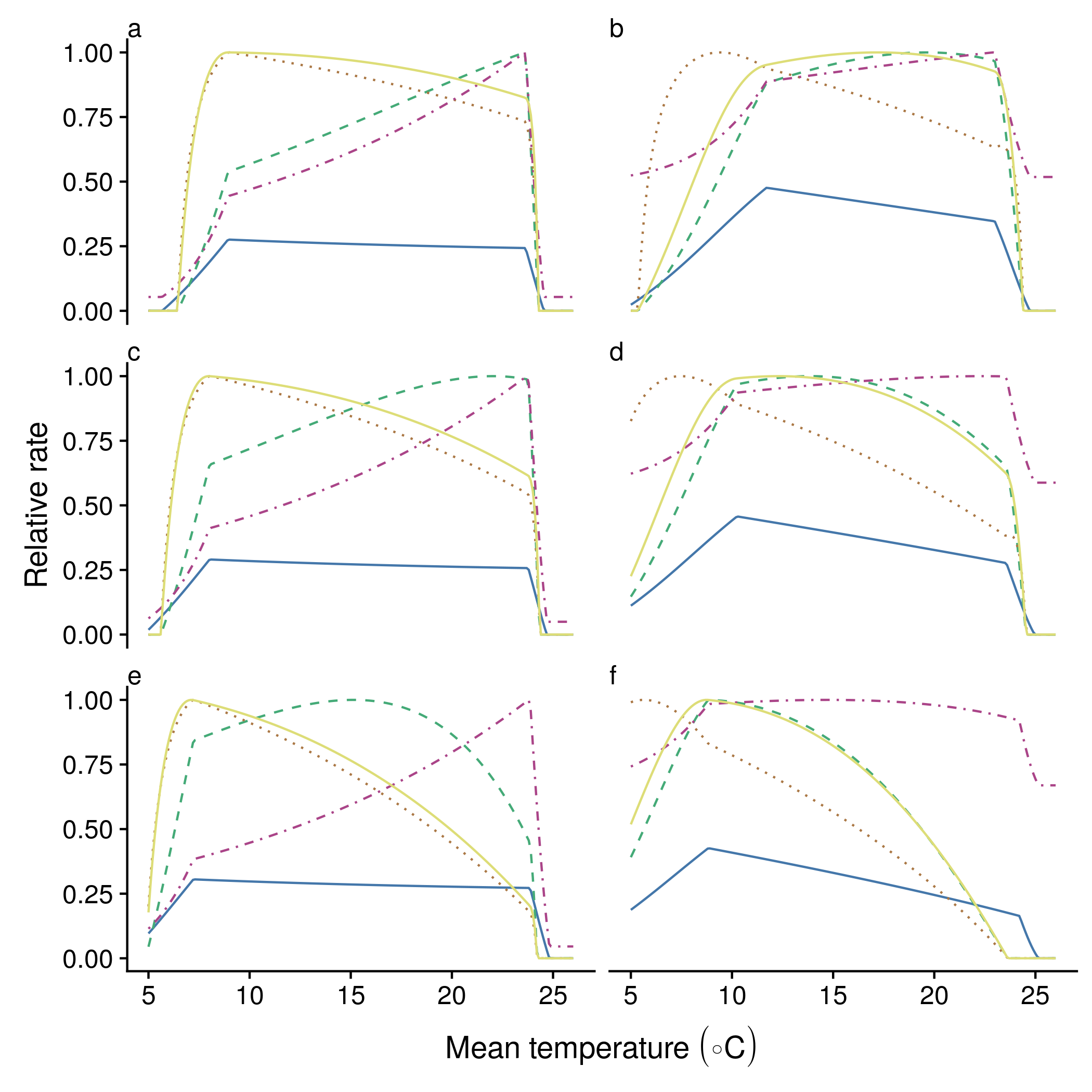

Supplement: FigureS5 [file figures5.png]

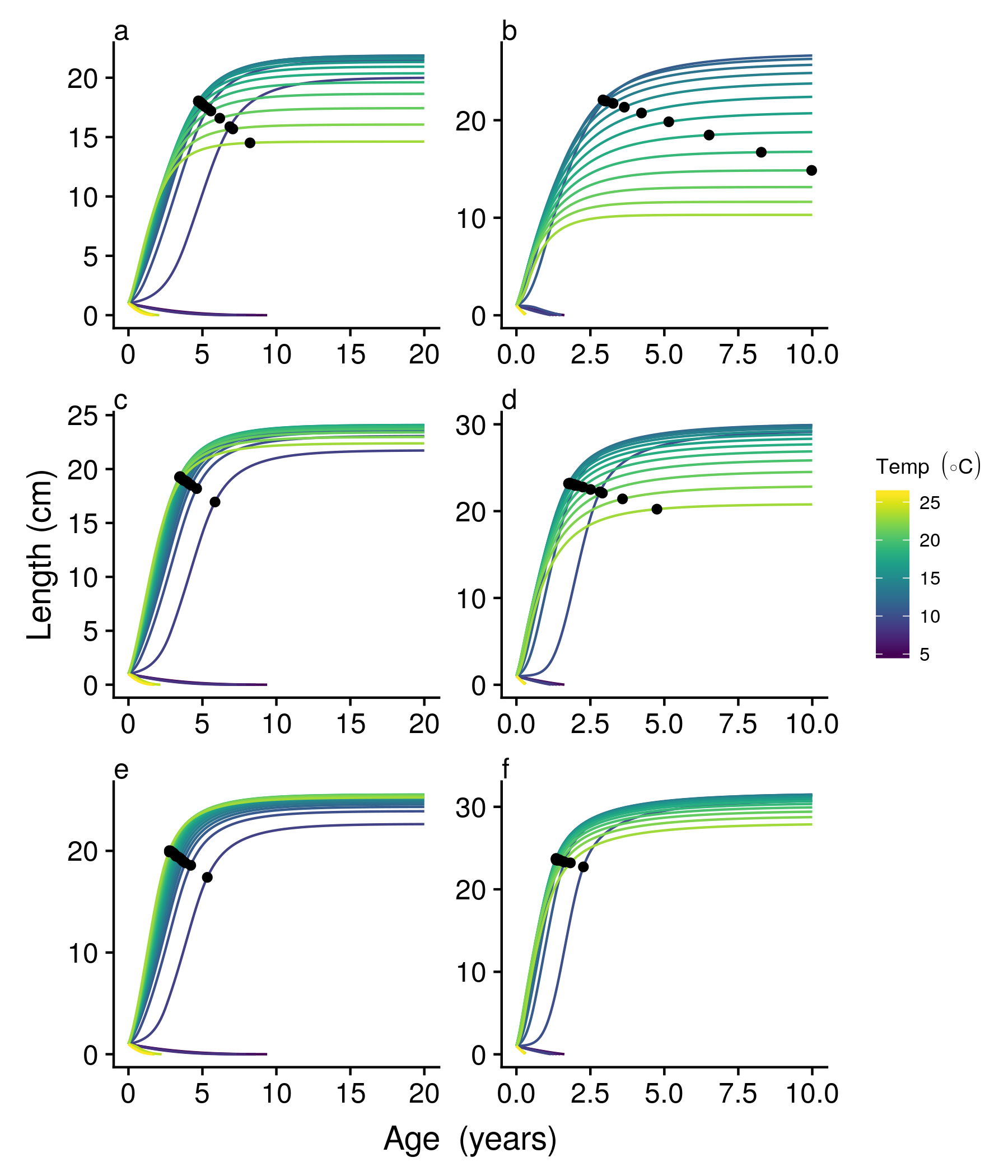

Supplement: FigureS6 [file figures6.png]

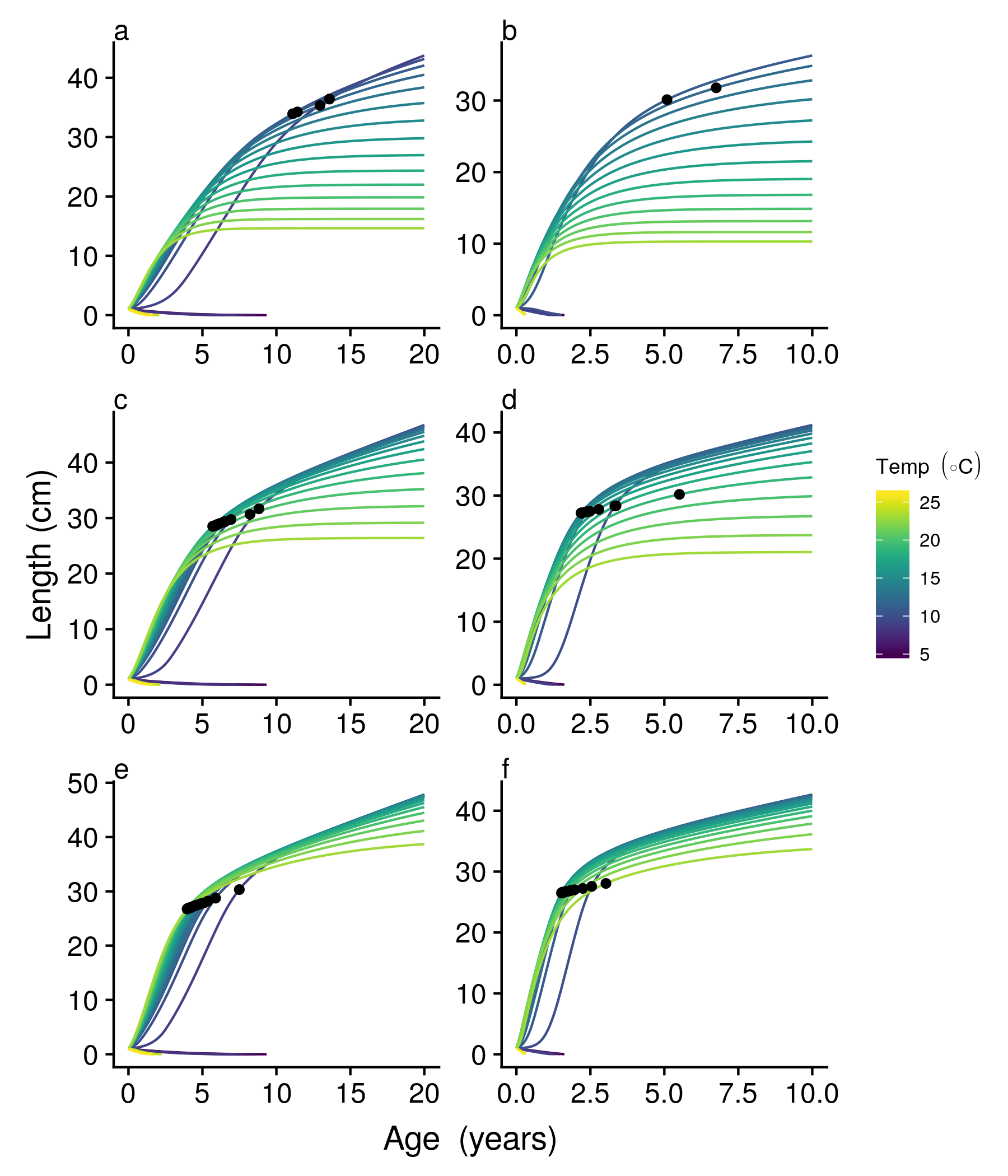

Supplement: FigureS7 [file figures7.png]
